# Supplementary material for: Emulating the Delivery of Sawtooth Proton Arc Therapy Plans on a Cyclotron-Based Proton Beam Therapy System
Source: Cancers (Basel). 2024 Sep 27;16(19):3315. doi: 10.3390/cancers16193315 (PMC11475827; doi:10.3390/cancers16193315)
Supplement: Supplementary file 1 [file cancers-16-03315-s001.zip › cancers-3153512-supplementary.pdf]

Supplementary material

1 Facility specific emulator delivery parameters

The Christie NHS Foundation Trust's proton beam therapy (PBT) facility has 3 clinical ProBeam® gantries (Varian: Palo Alto, CA, USA) and a separate Stoller Research Room (RR) with a fixed ProBeam® nozzle, all linked to a single cyclotron [1]. As each room shares the same beam-line energy selection system (ESS), we expect the energy layer switching times (ELSTs) to be constant throughout the facility. However, the variation in distance between each room and the cyclotron means the transmission efficiency to each room is different. Furthermore, differences in the electronics that process the readout from the Multi Strip Ionisation Chambers (MSIC) contained within the gantry nozzle means the RR and clinical gantries have individual monitor unit (MU) definitions. These two factors mean the MU delivery rate in the RR and clinical gantry will differ.

1.1 Energy layer switching times

The ESS at the Christie NHS Foundation Trust's PBT facility comprises of graphite wedges that are driven into the beam-line as well as an magnetic field based energy filtration system. Therefore due to magnetic hysteresis, we expect the ELSTs to be dependent on both starting energy and the magnitude of energy switch. Upward energy layer switches require a beam re-request and take 30 s regardless of this magnitude. In order to measure the downward ELSTs in both the RR and on a clinical gantry, a Tandem electrometer (PTW, Freiburg Germany) was used to record the cumulative charge measured by a Roos ionisation chamber (PTW, Freiburg Germany) with 50 ms precision. 1000 and 10 MUs were delivered using single spots in the RR and clinical gantry respectively. Once spot delivery was complete, the beam energy was decreased by the required amount to reach the next energy. This process was conducted once using energy switches of 1, 2, 4, 6, 8, 10 MeV in the RR at beam energies of 240 - 90 MeV in 30 MeV intervals. Measurements of switching times to reach the next corresponding beam energy were also taken. In each case, the temporal profile of measured charge was analysed to calculate the ELST with associated errors. Figure S1 shows there are significant differences in ELST measurements between the RR and clinical gantry and also between different starting energies.

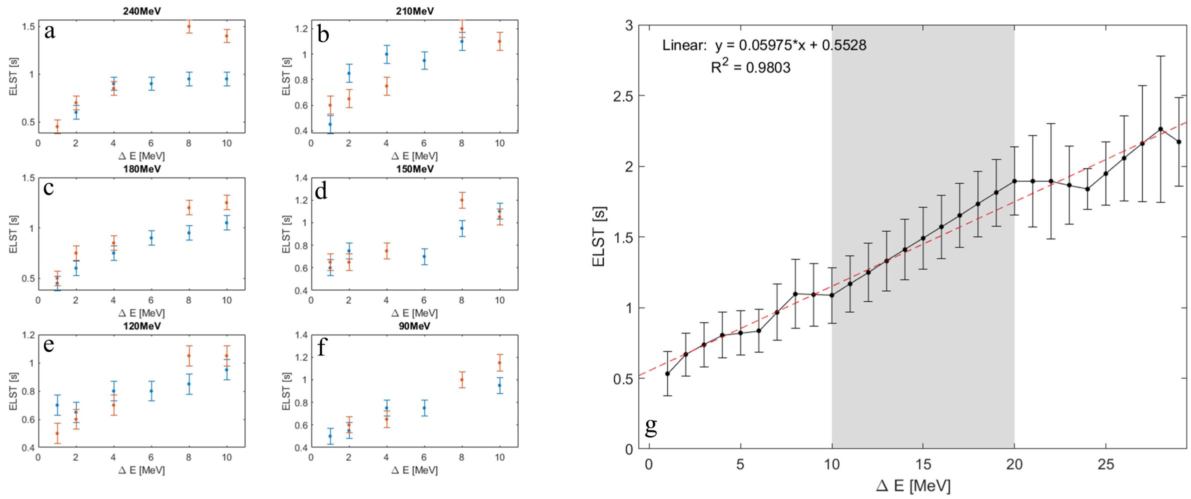

Figure S1: a)-f): Measurements of energy layer switching times (ELSTs) at the Christie Foundation Trust's PBT facility for a clinical gantry (orange) and the Stoller Research Room (blue). Individual starting beam energies shown in title. Errorbars indicate the associated uncertainty due to the electrometer temporal precision. g): Calculated ELST at each energy decrease from combining the measurements from the Stoller Research Room and clinical gantry across all energies. Errorbars outline the standard deviation of the 11-12 measurements underpinning this combination. Region of interpolation shaded in grey. Linear fit (dashed red line) shown with corresponding equation and R^2 value.

While the ELST generally increases relative to the magnitude of energy change, there is no clear

dependence on starting energy. Therefore, the clinical and RR ELST measurements were combined across all starting energies to develop a linear model of ELST relative to the magnitude of energy change, shown in figure S1. Linear interpolation was used to estimate the ELSTs in the range Δ E = 11 - 19 MeV.

1.2 MU delivery rates

MU delivery rates at each energy for the RR were calculate by measuring the delivery time of a 230 MeV, 10 x 10 cm spotmap under 2.5 mm spot spacing delivering 1 Gy at 1 nA nozzle current, allowing for the average MU delivery rate of each spot to be calculated. This was then scaled to different energies using the relative differences in transmission between the associated energy and 230 MeV. The MU delivery rate of the clinical gantry was then calculated as

$$\dot{\mathrm{MU}}_{\mathrm{clinic}}(E) = \dot{\mathrm{MU}}_{\mathrm{RR}}(E) \times \frac{T_{\mathrm{clinic}}(E)}{T_{\mathrm{RR}}(E)} \times \frac{(\#\mathrm{protons/MU})_{\mathrm{RR}}(E)}{(\#\mathrm{protons/MU})_{\mathrm{clinical}}(E)} \tag{S1}$$

where T corresponds to the transmission of each room and the number of protons per MU in each room was calculated using an in-house GATE-based Monte-Carlo (MC) system [2, 3]. The uncertainty associated with MU delivery rates was calculated as 8.98% by analysing cyclotron output current log files from July 2019 - Feb 2022 and taking the mean % standard deviation in the beam current fluctuations during each individual beam request across all requests during this period.

2 Emulated gantry motion during ependymoma E1 case

Figure S2 illustrates the emulated gantry angle each spot will be delivered at for the static IMPT, and single and dual arc PAT plans under the step-and-shoot and continuous delivery regimes. The magnified regions of these figures show the influence of continuous gantry rotation on the angle of spot delivery. As the gantry rotates during delivery of each energy layer, the spots within the energy layer are delivered at an angle further away from what was originally planned.

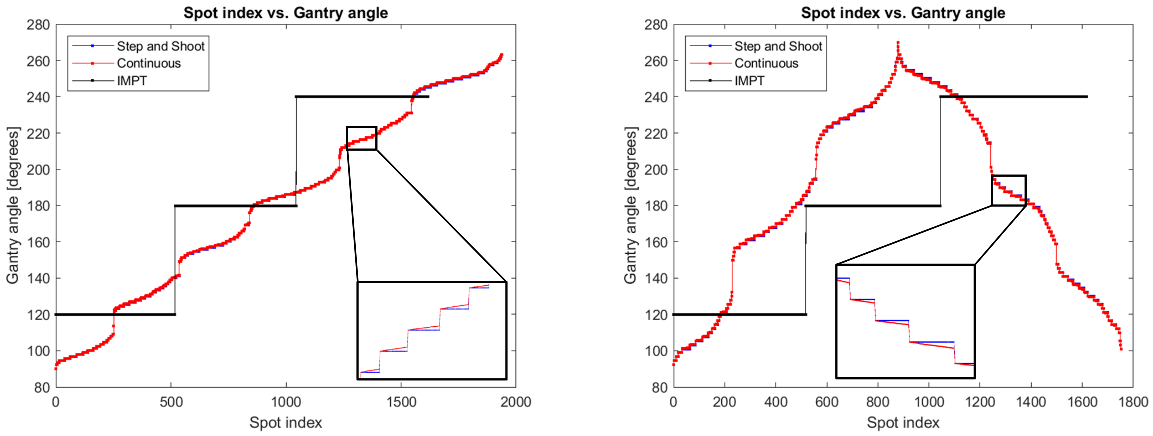

Figure S2: Emulated spot delivery angles for the single (left) and dual arc (right) Sawtooth PAT plans under the step-and-shoot and continuous delivery regimes on the ependymoma E1 case. Equivalent data from static IMPT plan emulation is also shown. Black box shows a magnification of the emulated data in highlighted region.

Figure S3 shows a breakdown of the emulated gantry velocity and acceleration during delivery of the single and dual arc PAT plans under the step-and-shoot and continuous delivery regimes, for the ependymoma E1 case. For PAT under continuous delivery, the angular position of each spot differs relative to step-and-shoot delivery depending on the angular spacing between energy layers and gantry motion. The greater this angular spacing, the greater the difference in the angle each spot is delivered at between the two delivery regimes. If this spacing is too large, this may cause significant deviations in the delivered dose distribution from what was originally planned.

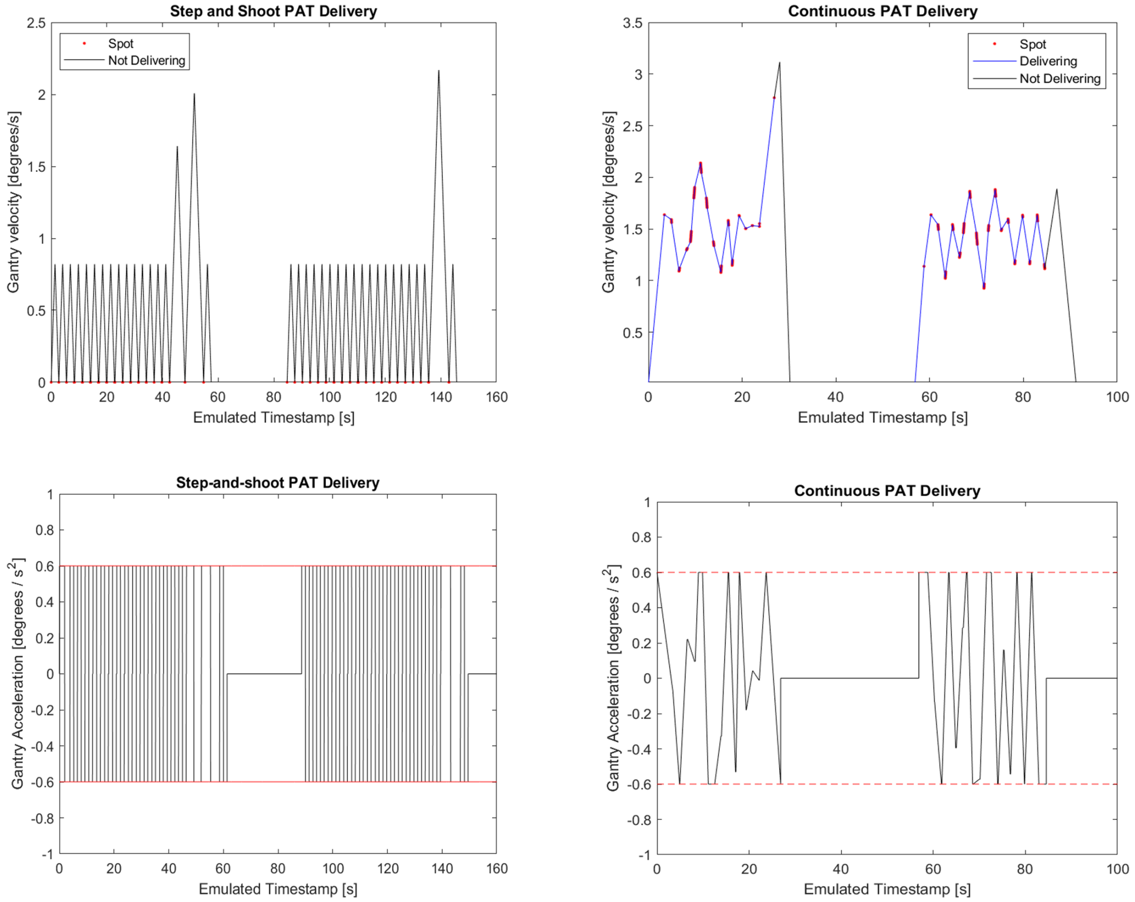

Figure S3: Emulated gantry velocity (top) and acceleration (bottom) vs timestamp under step-and-shoot (left) and continuous delivery (right) regimes for each spot (red dot) in the first two teeth of the 6-toothed single arc ependymoma E1 Sawtooth PAT plan. Blue and black lines indicate whether beam is delivering or not at any given point.

3 Further A1, B1, E1 and O1 results

3.1 Abdominal Phantom A1

After emulation, the 3 field IMPT plan was estimated to required 194.5 s to be delivered. PAT required a similar amount of time to scan between and deliver each spot compared to static IMPT. Additionally, the increased number of underlying energy layer switches meant both single and dual PAT plans had longer delivery times than static IMPT. Continuously delivered PAT plans slightly reduced the dead-time during delivery by 13% and 6% relative to static IMPT for single and dual arc PAT respectively. As such, the overall delivery time for continuously delivered single and dual arc PAT was approximately 29 and 37 seconds slower than static IMPT. Using step-and-shoot PAT delivery significantly increased the dead-time, such that it corresponded to 48% and 59% of the overall delivery time for single and dual arcs compared to 36% from static IMPT.

Figure S4 shows the dose distributions of the emulated single and dual arc PAT plans under continuous delivery, as well as the dose differences relative to the corresponding step-and-shoot plan. Under continuously delivery, the CTV $V_{95\%}/V_{105\%}$ remained consistent to within 1% relative to step-and-shoot delivery, with values of 100.0/0.2 $\%V_{CTV}$ and 100.0/0.01 $\%V_{CTV}$ for single and dual arc PAT plans respectively. Furthermore, the dose distributions from continuously delivered single and dual arc PAT plans showed a >99% and >97% pass-rate with the dose distributions resulting from step-and-shoot PAT delivery using a gamma analysis with dose-difference and distance-to-agreement criteria of 1% and 1 mm respectively. Across the 90° arc the mean and standard deviation of the differences in the angle of delivery between the planned and emulated spots was 0.33 ± 0.28° and 0.33 ± 0.43° for the non-perturbed and perturbed versions of the 4 toothed single arc where the control point spacing during planning was 0.8°. For the 4 toothed dual arc under 1.6° control point spacing, this difference was 0.65 ± 0.50° and 0.70 ± 0.53°. These differences caused shifts to the physical spot locations of 0.82 ± 2.52 mm and 1.16 ± 2.32 mm, and 1.07 ± 2.52 mm and 1.38 ± 2.81 mm for the single and dual arc respectively.

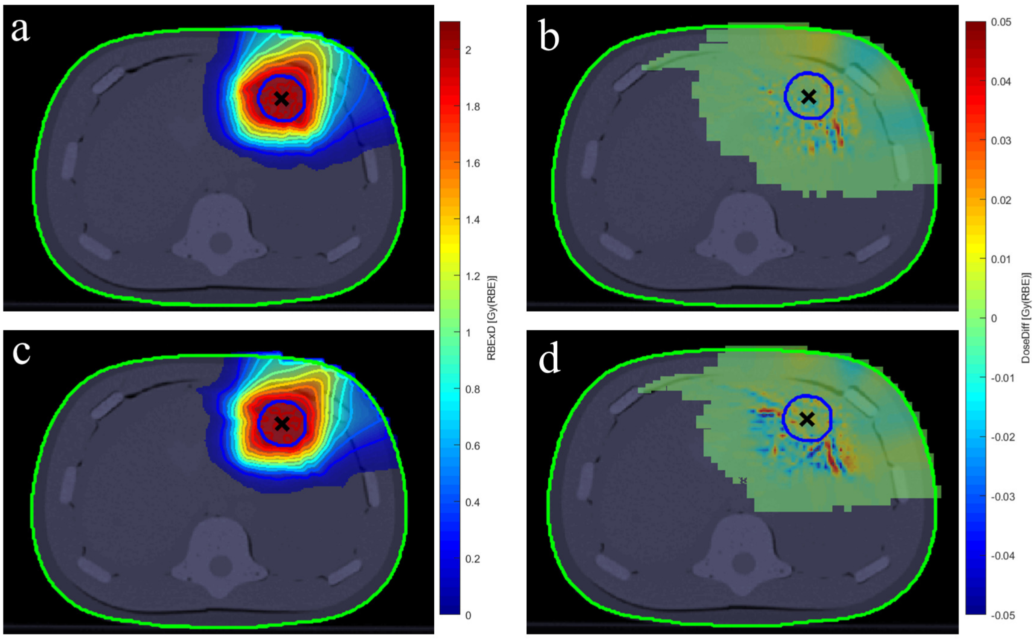

Figure S4: Single-fraction dose distribution of the abdominal phantom dataset under continuous single (a) and dual (c) arc PAT delivery. Dose differences of each plan relative to planned dose distribution of step-and-shoot PAT are shown in the right-hand column (b and d). CTV and external patient contours shown in blue and green respectively. Isocentre marked using black cross. Isodose lines shown in 10% and 5% intervals for 10-90% and 90-105% single-fraction dose respectively.

3.2 Ependymoma E1

After emulation, the 3 field IMPT plan was estimated to required approximately 120.9 s to be delivered. The overall delivery time for continuously delivered single and dual arc PAT was approximately 3.4 and 3.7 minutes slower than static IMPT. Despite single and dual arc PAT requiring 28% and 23% less time respectively than IMPT to deliver the MU's associated with the plan, the fact that PAT utilises a larger number of energy layers caused a 110% and 117% increase in cumulative energy layer switching time. Furthermore, despite continuous delivery requiring significantly less dead-time than step-and-shoot, 28% and 30% of the overall delivery time for single and dual arc PAT was limited by gantry motion. Across the 180° arc the mean and standard deviation of the differences in the angle of delivery between the planned and emulated spots was 0.42 ± 0.33^o and 0.41 ± 0.47^o for non-perturbed and perturbed versions of the 6 toothed single arc with a planning control spacing of 1.1^o. For the 6 toothed dual arc under 2.2^o control point spacing the differences were 0.86 ± 0.69^o and 0.90 ± 0.69^o. These differences caused shifts to the physical spot locations of 1.04 ± 1.75 mm and 1.47 ± 2.09 mm, and 1.55 ± 2.03 mm and 1.86 ± 2.18 mm for the single and dual arc respectively.

The clinical dose metrics across all PAT plans under continuous delivery were found to be within 1% of the step-and-shoot plan. As such, all emulated PAT plans were able to meet the corresponding clinical dose requirements for this treatment site. Across all relevant ROIs, a 100% pass-rate between the emulated and planned dose distributions was found from gamma analysis with dose-difference and distance-to-agreement criteria of 3% and 3 mm respectively. However, under the tighter criteria of 1% and 1 mm, 15% of brainstem voxels were found to have $\gamma > 1$ for the continuously delivered dual arc.

3.3 Chondrosarcoma B1

After emulation, the 4 field IMPT plan was estimated to required approximately 215.9 s to be delivered. The overall delivery time for continuously delivered single and dual arc PAT was approximately 4.6 and 7.4 minutes slower than static IMPT. Similar to the other cases, while static IMPT and Sawtooth PAT plans utilised similar spot scanning and delivery times, the energy switching time required to deliver PAT plans increased by 69% and 132% for single and dual arcs respectively. Additionally, unlike static IMPT, the delivery of PAT on current clinical systems was limited by gantry motion such that the overall delivery time was increased by at least 31% for both single and dual arc delivery. Step-and-shoot PAT delivery increased this dead-time further by between 3-7 minutes. Across the two 90° partial arcs the mean and standard deviation of the differences between the planned and emulated delivery angles was 0.25 ± 0.28^o and 0.24 ± 0.44^o for non-perturbed and perturbed versions of the 7 toothed single arc with 0.7^o control point spacing. For the 10 toothed dual arc with 1.0^o control point spacing the differences were 0.29 ± 0.31^o and 0.41 ± 0.36^o. These differences shifted the spots by 1.40 ± 2.80 mm and 2.12 ± 3.10 mm, and 1.50 ± 2.84 mm and 2.11 ± 3.21 mm for the single and dual arc respectively.

Figure S5 compares the single-fraction dose distribution of the planned (step-and-shoot) single arc PAT plans to those emulated under continuous delivery. In all PAT cases, the same percentage of dose metrics met the clinical thresholds in the nominal scenario after emulation. However, all PAT cases further reduced the dose to the primary and secondary CTVs, with $D_{95\%}$ and $D_{2\%}$ decreasing by 0.22 - 0.76% of the prescribed dose. Across all relevant ROIs, a 100% pass-rate between the emulated and planned dose distributions was found from gamma analysis with dose-difference and distance-to-agreement criteria of 3% and 3 mm respectively. Under the tighter criteria of 1%/1 mm, continuously delivered single arc PAT showed discrepancies of 8 - 13% in the CTVs and optic chiasm, while dual arc delivery showed 7-10% discrepancies in the CTVs and 8-24% in the cochleas.

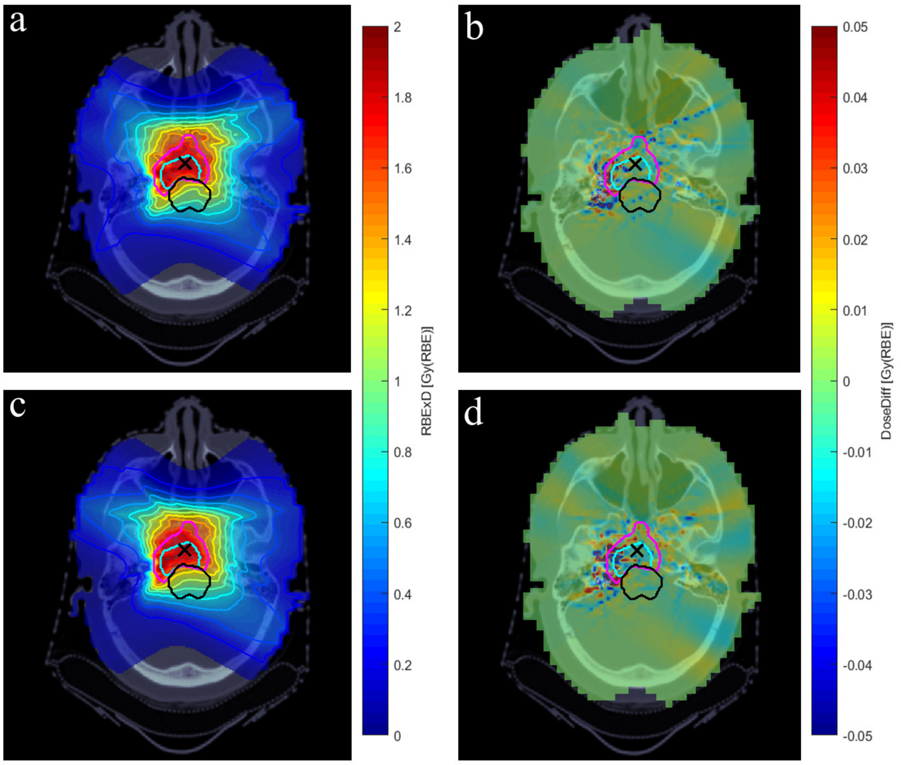

Figure S5: Single-fraction dose distribution of the chondrosarcoma B1 case under continuous single (a) and dual (c) arc PAT delivery. Dose differences of each plan relative to the planned dose distribution of step-and-shoot PAT are shown in the right-hand column (b and d). Primary and secondary CTVs, and brainstem patient contours shown in cyan, magenta and black respectively. Isocentre marked using black cross. Isodose lines shown in 10% and 5% intervals for 10-90% and 90-105% single-fraction dose respectively.

3.4 Oropharyngeal O1

After emulation, the 5 field IMPT plan was estimated to required approximately 447.5 s to be delivered. The overall delivery time for continuously delivered single and dual arc PAT was approximately 4.9 and 1.5 minutes slower than static IMPT. As with the previous cases, all emulated PAT plans required an amount of time to scan and deliver the MU's associated with each spot that was consistent with static IMPT delivery. However, in this case since the dual arc PAT plan only had one additional tooth relative to the number of fields in the static IMPT plan (6 vs 5), both plans were consistent in the total ELST required during delivery. As the single arc PAT plan utilised 8 teeth during delivery, the total ELST required was $\sim$48% longer than static IMPT or dual arc PAT. The dead-time resulting from gantry motion was at least 190% and 85% larger for continuously delivered single and dual arc PAT relative to static IMPT, with step-and-shoot PAT increasing this further by at least 51%. Across the 180° arc the mean and standard deviation of the differences between the planned and emulated delivery angles was $0.26 \pm 0.19^{\circ}$ and $0.26 \pm 0.28^{\circ}$ for non-perturbed and perturbed versions of the 8 toothed single arc with 0.6° control point spacing. For the 6 toothed dual arc with 1.6° control point spacing the differences were $0.81 \pm 0.59^{\circ}$ and $0.86 \pm 0.62^{\circ}$. These differences shifted the spots by 0.96 ± 2.05 mm and 1.48 ± 2.37 mm, and 1.78 ± 2.44 mm and 2.10 ± 2.59 mm for the single and dual arc respectively.

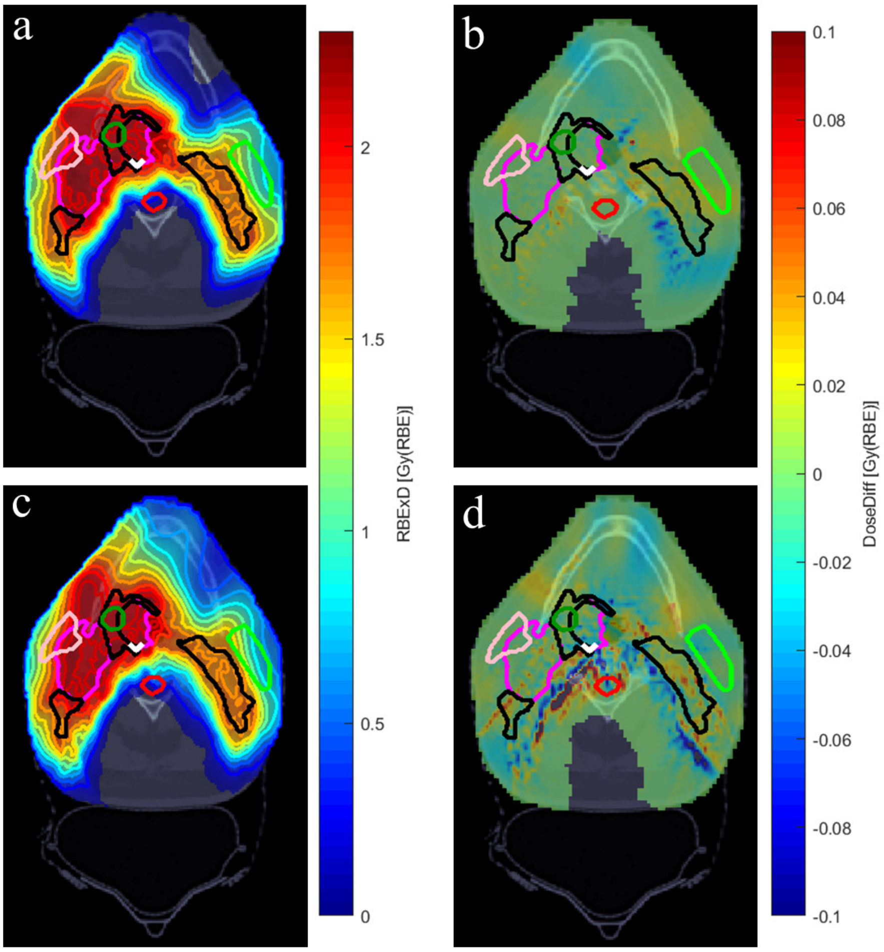

Figure S6: Single-fraction dose distribution of the oropharyngeal O1 case under continuous single (a) and dual (c) arc PAT delivery. Dose differences of each plan relative to the planned dose distribution of step-and-shoot PAT are shown in the right-hand column (b and d). Primary and secondary CTV, spinal cord, ipsilateral and contralateral parotid and ipsilateral sub-mandibular contours are shown in magenta, black, red, pink, light green and dark green respectively. Isocentre (x,y) location marked by white arrowhead. Isodose lines shown in 10% and 5% intervals for 10-90% and 90-105% single-fraction dose respectively.

Figure S6 compares the single-fraction dose distribution of the planned (step-and-shoot) single arc PAT plans to those emulated under continuous delivery. All relevant ROIs pass the clinical dose metrics in the nominal scenario of all emulated PAT plans, except the contralateral sub-mandibular gland. Here the single-fraction D_{mean} increased from 11.4 Gy to 29.2 Gy after emulation in the case of the single arc plan but remains the same for dual arc delivery. Secondary CTV coverage ($D_{5\%}$) also decreased by 5.8 - 6.1% of the prescribed dose across single and dual arc delivery but remains of clinical standard. Across all relevant ROIs, a 100% pass-rate between the emulated and planned dose distributions were found from gamma analysis with dose-difference and distance-to-agreement criteria of 3% and 3 mm respectively. Under the tighter criteria of 1%/1 mm, single arc therapy showed a ¿97% pass-rate in all ROIs except the contralateral sub-mandibular gland with a 96% pass-rate. Dual arc delivery without the application of random spot permutations showed worse agreement, with a 69% pass-rate in the contralateral sub-mandibular gland, 93% in the primary CTV, and ~81% in the secondary CTV, brainstem and spinal cord.

4 Additional datasets

4.1 Emulated delivery times

4.1.1 Ependymoma E2

Table S1: Breakdown of emulated time spent on each part of delivery for 4 field static IMPT plan and 4 tooth single and dual arc PAT plan under step-and-shoot and continuous delivery regimes for the ependymoma E2 dataset on a clinical gantry. Errors correspond to one standard deviation deriving from variations in the experimental measurements of ELST and MU delivery rates are shown. Abbreviations: EL = energy layer, SAS = step-and-shoot, CON = continuous.

Delivery Method		EL switching	Spot scanning	Spot delivery	Dead-time	Total
Static IMPT		119.0 ± 10.2	4.5	39.1 ± 3.5	74.3	237.0 ± 13.7
PAT single arc	SAS	165.7 ± 13.1	6.0	38.0 ± 3.4	217.9	427.6 ± 16.5
	CON				80.9	290.6 ± 16.5
PAT dual arc	SAS	172.0 ± 14.2	6.3	35.4 ± 3.2	350.7	564.4 ± 17.4
	CON				92.3	304.0 ± 17.4

4.1.2 Chondrosarcoma B2

Table S2: Breakdown of the emulated time spent on each part of delivery for 4 field static IMPT plan and 7 tooth single and 8 tooth dual partial arc PAT plan under step-and-shoot and continuous delivery regimes on a clinical gantry for the chondrosarcoma B2 case. Errors correspond to one standard deviation deriving from variations in the experimental measurements of ELST and MU delivery rates are shown. Abbreviations: EL = energy layer, SAS = step-and-shoot, CON = continuous.

Delivery Method		EL switching	Spot scanning	Spot delivery	Dead-time	Total
Static IMPT		188.8 ± 17.1	7.4	52.6 ± 4.7	65.0	313.9 ± 21.8
PAT single arc	SAS	323.7 ± 24.9	6.2	42.8 ± 3.8	364.6	737.2 ± 28.7
	CON				157.6	530.3 ± 28.7
PAT dual arc	SAS	336.5 ± 21.9	8.0	41.5 ± 3.7	584.2	970.2 ± 25.6
	CON				149.4	535.4 ± 25.6

4.1.3 Oropharyngeal O2

Table S3: Breakdown of the emulated time spent on each part of delivery for 5 field static IMPT plan and 6 tooth single and 8 tooth dual partial arc PAT plan under step-and-shoot and continuous delivery regimes on a clinical gantry for the oropharyngeal O2 case. Errors correspond to one standard deviation deriving from variations in the experimental measurements of ELST and MU delivery rates are shown. Abbreviations: EL = energy layer, SAS = step-and-shoot, CON = continuous.

Delivery Method		EL switching	Spot scanning	Spot delivery	Dead-time	Total
Static IMPT		255.1 ± 23.4	11.5	136.7 ± 12.3	61.7	465.0 ± 35.7
PAT single arc	SAS	285.5 ± 23.5	10.8	131.4 ± 11.8	351.2	777.9 ± 35.3
	CON				145.2	571.8 ± 35.3
PAT dual arc	SAS	393.1 ± 31.7	13.8	140.4 ± 12.6	661.4	1208.7 ± 44.3
	CON				187.0	734.3 ± 44.3

4.2 Emulated dose distributions

4.2.1 Ependymoma E2

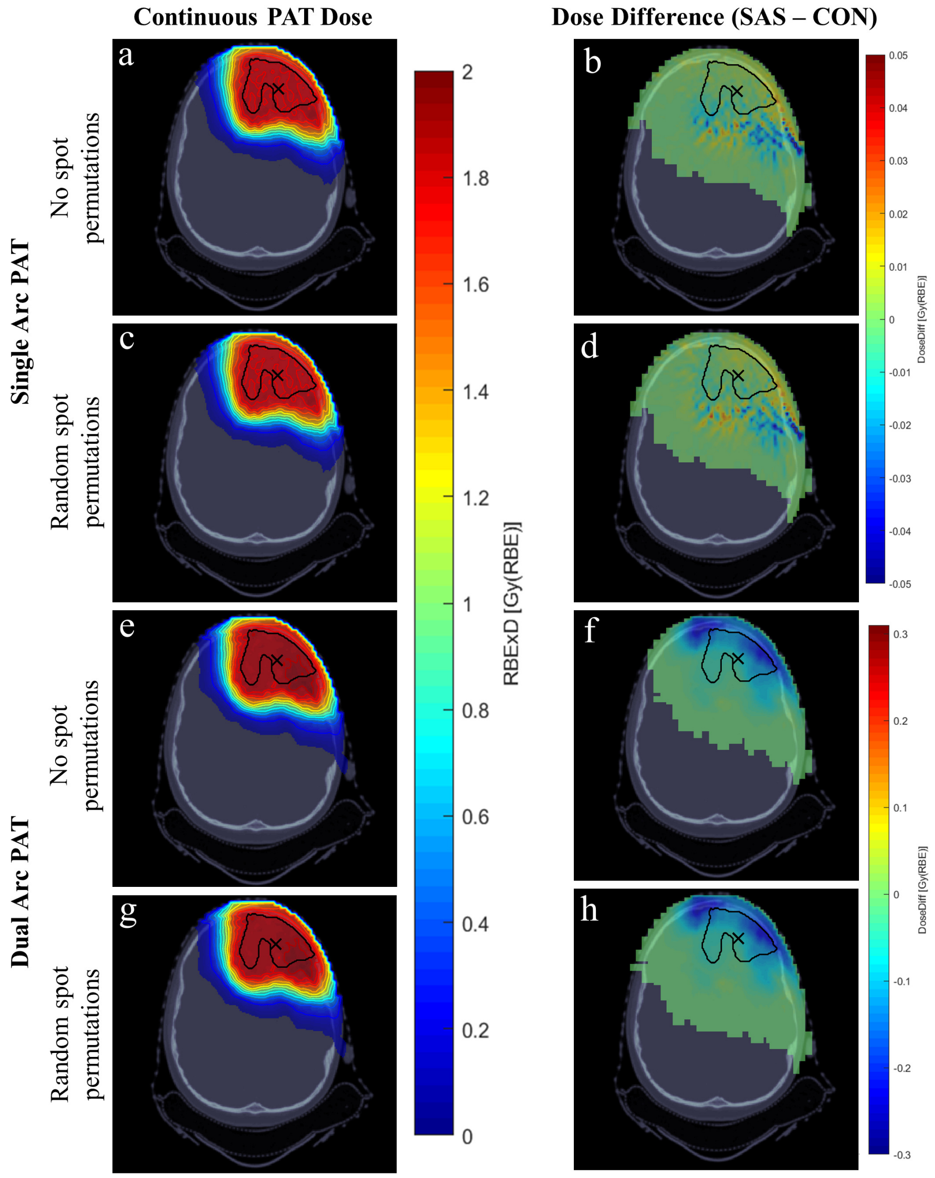

Figure S7: Single-fraction dose distribution of the ependymoma E2 case under continuous single (a) and dual (e) arc PAT delivery. The dose distribution resulting from applying random shifts to the spot positions and gantry angles of these plans are also shown for single (c) and dual (g) arc delivery. These shifts were drawn in accordance with a Gaussian probability distribution with $\sigma = 0.33$ mm/°. Dose differences of each plan relative to the planned dose distribution of step-and-shoot PAT are shown in the right-hand column (b,d,f and h). CTV contour shown in black. Isocentre marked using black cross. Isodose lines shown in 10% and 5% intervals for 10-90% and 90-105% single-fraction dose respectively.

4.2.2 Chondrosarcoma B2

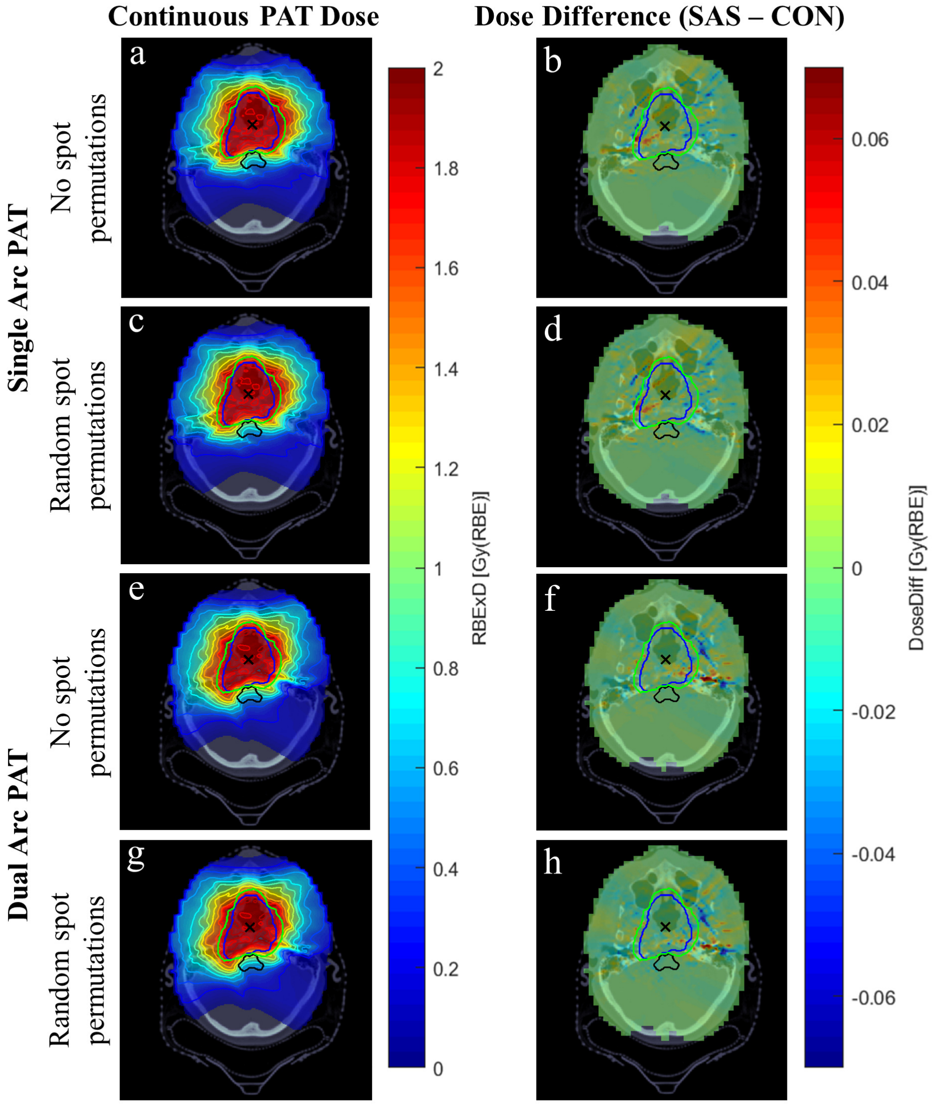

Figure S8: Single-fraction dose distribution of the chondrosarcoma case B2 under continuous single (a) and dual (e) arc PAT delivery. The dose distribution resulting from applying random shifts to the spot positions and gantry angles of these plans are also shown for single (c) and dual (g) arc delivery. These shifts were drawn in accordance with a Gaussian probability distribution with $\sigma = 0.33$ mm/°. Dose differences of each plan relative to the planned dose distribution of step-and-shoot PAT are shown in the right-hand column (b,d,f and h). CTV1, CTV2 and brainstem patient contours shown in blue, green and black respectively. Isocentre marked using black cross. Isodose lines shown in 10% and 5% intervals for 10-90% and 90-105% single-fraction dose respectively.

4.2.3 Oropharyngeal O2

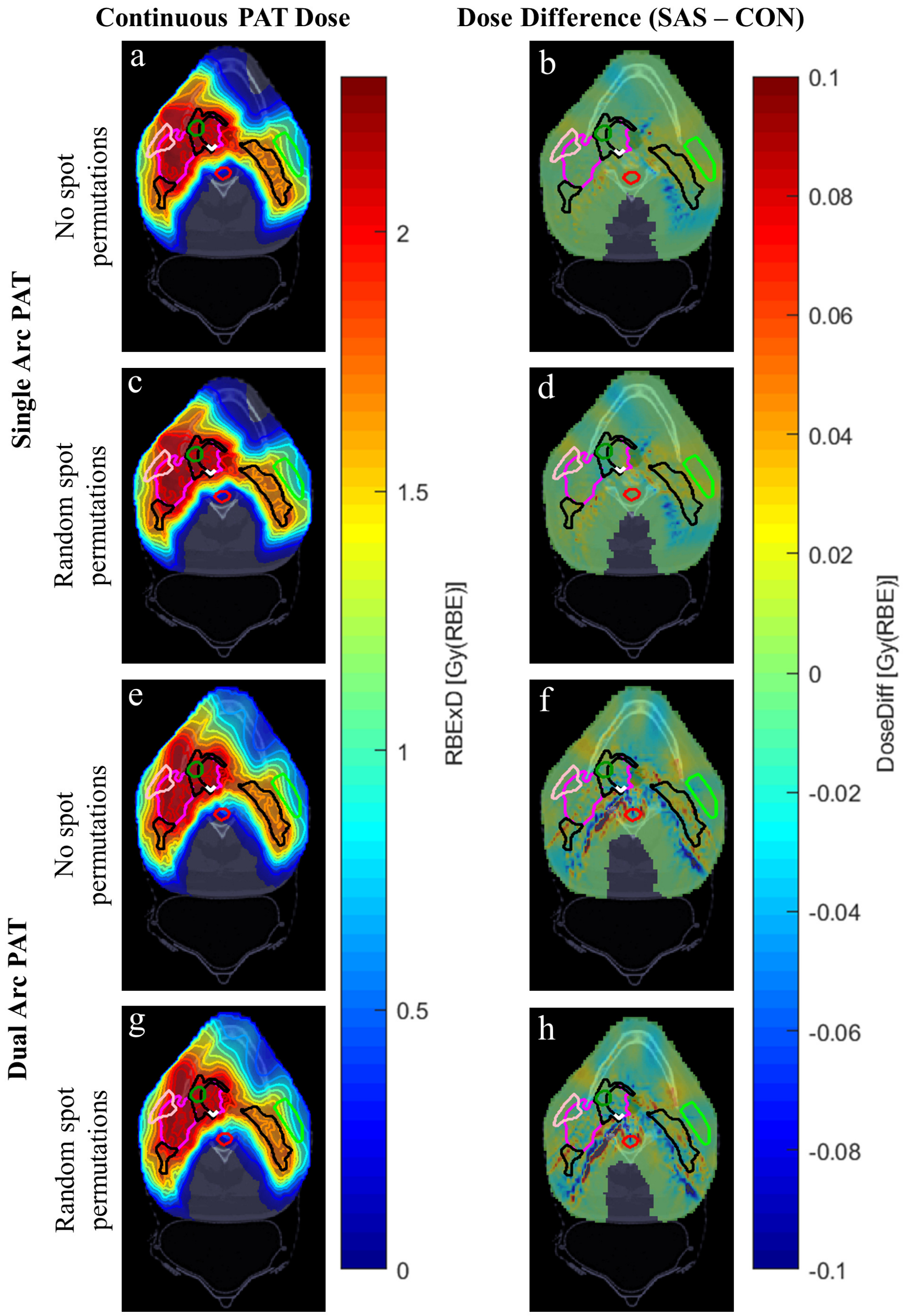

Figure S9: Single-fraction dose distribution of the oropharyngeal case O2 under continuous single (a) and dual (e) arc PAT delivery. The dose distribution resulting from applying random shifts to the spot positions and gantry angles of these plans are also shown for single (c) and dual (g) arc delivery. These shifts were drawn in accordance with a Gaussian probability distribution with $\sigma = 0.33$ mm/°. Dose differences of each plan relative to the planned dose distribution of step-and-shoot PAT are shown in the right-hand column (b,d,f and h). Primary and secondary CTVs, spinal cord, ipsilateral and contralateral parotid contours shown in blue, black, red, green and pink respectively. Isocentre (x,y) location marked by white arrowhead. Isodose lines shown in 10% and 5% intervals for 10-90% and 90-105% single-fraction dose respectively.

4.3 Clinical dose metrics & gamma analysis data

4.3.1 Ependymoma E1

Table S4: Comparison of the dose metric change in the nominal scenario during each emulated delivery technique relative to the original step-and-shoot PAT plan for the ependymoma E1 dataset. Abbreviations: CTV = clinical target volume, L = left, R = right.

ROI	Metric	Clinical goal	Single arc PAT		Dual arc PAT		Units
			Original	Shifted	Original	Shifted	
CTV	$V_{95\%}$	> 98	-0.78	-0.69	-0.17	-0.17	$\%V_{CTV}$
	$V_{105\%}$	< 10	0.00	0.00	0.00	0.00	$\%V_{CTV}$
	ΔD	—	0.00	0.00	0.01	0.01	Gy
Brainstem	$D_{1\%}$	< 59.4	0.00	-0.00	0.00	0.00	Gy
	$D_{10\%}$	< 58	-0.00	0.00	-0.01	-0.00	Gy
	$D_{50\%}$	< 56	-0.02	-0.02	-0.01	-0.01	Gy
Spinal cord	$D_{2\%}$	≤ 54	-0.01	-0.01	-0.02	-0.02	Gy
Cochlea L	D_{mean}	< 30	-0.00	0.00	0.00	-0.00	Gy
Cochlea R	D_{mean}	< 30	-0.00	0.00	0.00	-0.00	Gy

Table S5: Percentage pass rates of voxels within each ROI from the ependymoma case E1 under gamma analysis comparing the dose distributions from single and dual arc PAT under step-and-shoot delivery to the corresponding plan under continuous delivery with and without applying the random angular and positional shifts from a Gaussian probability distribution with $\sigma = 0.33$ mm/° to each spot. Abbreviations: DD = dose-difference, DTA = distance-to-agreement, CTV = clinical target volume, L = Left, R = Right.

ROI	DD/DTA Criteria	Single arc PAT		Dual arc PAT	
		Original	Shifted	Original	Shifted
CTV	1%/1mm	99.5	99.1	95.7	94.8
	3%/3mm	100.0	100.0	100.0	100.0
Brainstem	1%/1mm	96.7	96.2	85.2	85.9
	3%/3mm	100.0	100.0	100.0	100.0
Spinal cord	1%/1mm	100.0	100.0	100.0	100.0
	3%/3mm	100.0	100.0	100.0	100.0
Cochlea L	1%/1mm	100.0	100.0	100.0	100.0
	3%/3mm	100.0	100.0	100.0	100.0
Cochlea R	1%/1mm	100.0	100.0	100.0	100.0
	3%/3mm	100.0	100.0	100.0	100.0
Optic Chiasm	1%/1mm	100.0	100.0	100.0	100.0
	3%/3mm	100.0	100.0	100.0	100.0
Patient	1%/1mm	99.0	98.9	96.7	96.7
	3%/3mm	100.0	100.0	100.0	100.0

4.3.2 Ependymoma E2

Table S6: Comparison of the dose metric change in the nominal scenario during each emulated delivery technique relative to the original step-and-shoot PAT plan for the ependymoma E2 dataset. Bold text indicates a failure of clinical tolerance. Abbreviations: CTV = clinical target volume, ON = optic nerve, LG = lacrimal gland, L = left.

ROI	Metric	Clinical goal	Single arc PAT		Dual arc PAT		Units
			Original	Shifted	Original	Shifted	
CTV	$V_{95\%}$	> 98	-0.07	-0.07	0.46	0.46	$\%V_{CTV}$
	$V_{105\%}$	< 10	0.00	0.00	**17.99**	**17.88**	$\%V_{CTV}$
	ΔD	—	0.00	0.00	0.14	0.15	Gy
Eye L	$D_{10\%}$	< 54	0.00	0.00	-0.09	-0.09	Gy
	$D_{50\%}$	< 30	0.00	0.00	-0.04	-0.04	Gy
	$D_{90\%}$	< 10	0.00	0.00	-0.01	-0.01	Gy
ON L	D_{mean}	< 54	0.00	0.00	-0.04	-0.04	Gy
ON R	D_{mean}	< 54	0.00	0.00	-0.04	-0.04	Gy
OC	D_{max}	< 54	-0.02	-0.02	-0.01	-0.01	Gy
LG L	D_{mean}	< 41	0.00	0.00	-0.06	-0.06	Gy

Table S7: Percentage pass rates of voxels within each ROI from the ependymoma E2 case under gamma analysis comparing the dose distributions from single and dual arc PAT under step-and-shoot delivery to the corresponding plan under continuous delivery with and without applying the random angular and positional shifts from a Gaussian probability distribution with $\sigma = 0.33$ mm/° to each spot. Abbreviations: DD = dose-difference, DTA = distance-to-agreement, CTV = clinical target volume, ON = optic nerve, LG = lacrimal gland, L = left.

ROI	DD/DTA Criteria	Single arc PAT		Dual arc PAT	
		Original	Shifted	Original	Shifted
CTV	1%/1mm	99.9	99.9	9.5	9.5
	3%/3mm	100.0	100.0	37.4	37.0
Brainstem	1%/1mm	100.0	100.0	100.0	100.0
	3%/3mm	100.0	100.0	100.0	100.0
Eye L	1%/1mm	100.0	100.0	59.6	58.7
	3%/3mm	100.0	100.0	100.0	100.0
LG L	1%/1mm	100.0	100.0	86.7	85.1
	3%/3mm	100.0	100.0	100.0	100.0
Patient	1%/1mm	98.3	98.4	49.2	49.1
	3%/3mm	100.0	100.0	85.8	85.7

4.3.3 Chondrosarcoma B1

Table S8: Comparison of the dose metric change in the nominal scenario during each emulated delivery technique relative to the original step-and-shoot PAT plan for the chondrosarcoma B1 dataset. Bold text indicates a failure of clinical tolerance. Abbreviations: CTV = clinical target volume, ON = optic nerve, OC = optic chiasm, L = left, R = right.

ROI	Metric	Clinical goal	Single arc PAT		Dual arc PAT		Units
			Original	Shifted	Original	Shifted	
CTV1	$D_{98\%}$	> 90	**-0.22**	**-0.33**	**-0.31**	**-0.09**	$\%D_{pres}$
	$D_{95\%}$	> 95	**-0.37**	**-0.52**	**-0.22**	**-0.26**	$\%D_{pres}$
	$D_{2\%}$	< 110	-0.31	-0.76	-0.30	-0.68	$\%D_{pres}$
	ΔD	—	-0.02	-0.02	0.02	0.02	Gy
CTV2	$D_{98\%}$	> 90	-0.81	-0.53	-1.10	-1.34	$\%V_{CTV2}$
	$D_{95\%}$	> 95	-0.62	-0.49	-0.91	-1.11	$\%V_{CTV2}$
	ΔD	—	-0.03	-0.03	-0.01	-0.02	Gy
OC	$D_{2\%}$	< 60	-0.01	-0.01	0.00	-0.01	Gy
	D_{mean}	< 54	-0.01	-0.01	0.00	0.00	Gy
ON L	$D_{2\%}$	< 60	0.00	0.00	0.00	0.00	Gy
	D_{mean}	< 54	0.00	0.00	0.00	0.00	Gy
ON R	$D_{2\%}$	< 60	0.00	0.00	0.01	0.01	Gy
	D_{mean}	< 54	0.00	0.00	0.00	0.00	Gy
Brainstem	D_2	< 60	0.00	0.00	0.00	0.00	Gy
Spinal cord	D_{max}	< 67	0.01	0.01	0.00	0.00	Gy
Cochlea L	D_{mean}	< 45	-0.01	-0.01	0.01	0.02	Gy
Cochlea R	D_{mean}	< 45	0.00	0.00	-0.01	-0.01	Gy

Table S9: Percentage pass rates of voxels within each ROI from the chondrosarcoma B1 case under gamma analysis comparing dose distributions from single and dual arc PAT under step-and-shoot delivery to the corresponding plan under continuous delivery with and without applying the random angular and positional shifts from a Gaussian probability distribution with σ = 0.33 mm/° to each spot. Abbreviations: DD = dose-difference, DTA = distance-to-agreement, CTV = clinical target volume, ON = optic nerve, OC = optic chiasm, L = left, R = right.

ROI	DD/DTA Criteria	Single arc PAT		Dual arc PAT	
		Original	Shifted	Original	Shifted
CTV1	1%/1mm	87.1	86.5	92.5	92.7
	3%/3mm	100.0	100.0	100.0	100.0
CTV2	1%/1mm	89.5	89.3	91.0	90.1
	3%/3mm	100.0	100.0	100.0	100.0
OC	1%/1mm	90.8	91.8	100.0	98.4
	3%/3mm	100.0	100.0	100.0	100.0
ON L	1%/1mm	99.0	99.3	99.9	99.7
	3%/3mm	100.0	100.0	100.0	100.0
ON R	1%/1mm	100.0	100.0	99.6	99.2
	3%/3mm	100.0	100.0	100.0	100.0
Brainstem	1%/1mm	98.7	98.8	99.9	99.8
	3%/3mm	100.0	100.0	100.0	100.0
Spinal cord	1%/1mm	100.0	100.0	100.0	100.0
	3%/3mm	100.0	100.0	100.0	100.0
Cochlea L	1%/1mm	100.0	100.0	86.1	92.1
	3%/3mm	100.0	100.0	100.0	100.0
Cochlea R	1%/1mm	93.2	95.1	80.6	76.7
	3%/3mm	100.0	100.0	100.0	100.0
Patient	1%/1mm	99.2	99.2	99.3	99.2
	3%/3mm	100.0	100.0	100.0	100.0

4.3.4 Chondrosarcoma B2

Table S10: Comparison of the dose metric change in the nominal scenario during each emulated delivery technique relative to the original step-and-shoot PAT plan for the chondrosarcoma B2 dataset. Bold text indicates a failure of clinical tolerance. Abbreviations: CTV = clinical target volume, ON = optic nerve, OC = optic chiasm, L = left, R = right.

ROI	Metric	Clinical goal	Single arc PAT		Dual arc PAT		Units
			Original	Shifted	Original	Shifted	
CTV1	$D_{98\%}$	> 90	**0.47**	**0.62**	**0.12**	**0.17**	$\%D_{pres}$
	$D_{95\%}$	> 95	**0.02**	**0.19**	**0.00**	**-0.06**	$\%D_{pres}$
	$D_{2\%}$	< 110	**0.62**	**0.33**	0.00	**-0.15**	$\%D_{pres}$
	ΔD	—	0.00	0.00	0.00	-0.01	Gy
CTV2	$D_{98\%}$	> 90	**-0.83**	**-0.84**	**0.47**	**0.92**	$\%V_{CTV2}$
	$D_{95\%}$	> 95	**0.30**	**0.49**	**-0.16**	**-0.31**	$\%V_{CTV2}$
	ΔD	—	0.00	-0.01	0.00	0.00	Gy
OC	$D_{2\%}$	< 60	0.00	-0.01	-0.01	-0.01	Gy
	D_{mean}	< 54	0.00	0.00	0.00	0.00	Gy
ON L	$D_{2\%}$	< 60	0.02	0.01	-0.01	0.00	Gy
	D_{mean}	< 54	0.02	0.02	0.00	0.00	Gy
ON R	$D_{2\%}$	< 60	0.00	0.01	-0.09	-0.01	Gy
	D_{mean}	< 54	0.00	-0.01	0.00	0.00	Gy
Brainstem	D_2	< 63	0.00	0.00	0.00	0.00	Gy
Spinal cord	D_{max}	< 67	0.02	0.02	-0.01	0.00	Gy
Cochlea L	D_{mean}	< 45	0.00	0.01	0.02	0.03	Gy
Cochlea R	D_{mean}	< 45	**-0.01**	**0.00**	**0.01**	**0.01**	Gy

Table S11: Percentage pass rates of voxels within each ROI from the chondrosarcoma B2 case under gamma analysis comparing dose distributions from single and dual arc PAT under step-and-shoot delivery to the corresponding plan under continuous delivery with and without applying the random angular and positional shifts from a Gaussian probability distribution with σ = 0.33 mm/° to each spot. Abbreviations: DD = dose-difference, DTA = distance-to-agreement, CTV = clinical target volume, ON = optic nerve, OC = optic chiasm, L = left, R = right.

ROI	DD/DTA Criteria	Single arc PAT		Dual arc PAT	
		Original	Shifted	Original	Shifted
CTV1	1%/1mm	87.4	87.2	97.8	97.5
	3%/3mm	99.5	99.5	100.0	100.0
CTV2	1%/1mm	87.4	88.1	97.3	97.1
	3%/3mm	99.7	99.6	100.0	100.0
OC	1%/1mm	99.4	99.8	100.0	100.0
	3%/3mm	100.0	100.0	100.0	100.0
ON L	1%/1mm	38.9	37.2	98.6	98.8
	3%/3mm	100.0	100.0	100.0	100.0
ON R	1%/1mm	73.8	73.6	98.0	96.1
	3%/3mm	100.0	100.0	100.0	100.0
Brainstem	1%/1mm	99.9	99.9	100.0	99.7
	3%/3mm	100.0	100.0	100.0	100.0
Spinal cord	1%/1mm	100.0	96.5	100.0	100.0
	3%/3mm	100.0	100.0	100.0	100.0
Cochlea L	1%/1mm	100.0	95.2	69.0	54.8
	3%/3mm	100.0	100.0	100.0	100.0
Cochlea R	1%/1mm	93.2	92.0	100.0	96.6
	3%/3mm	100.0	100.0	100.0	100.0

4.3.5 Oropharyngeal O1

Table S12: Comparison of the dose metric change in the nominal scenario during each emulated delivery technique relative to the original step-and-shoot PAT plan for the oropharyngeal O1 dataset. Abbreviations: CTV = clinical target volume, SMG = sub-mandibular gland, Ip = ipsilateral, Con = contralateral, ALARP = as low as reasonably possible.

ROI	Metric	Clinical goal	Single arc PAT		Dual arc PAT		Units
			Original	Shifted	Original	Shifted	
CTV1	$D_{99\%}$	> 90	0.05	-0.13	-0.14	-0.18	$\%D_{pres}$
	$D_{95\%}$	> 95	0.00	-0.19	-0.16	-0.06	$\%D_{pres}$
	$D_{5\%}$	≤ 105	-0.08	-0.18	0.16	0.28	$\%D_{pres}$
	$D_{2\%}$	≤ 107	-0.09	-0.20	0.28	0.52	$\%D_{pres}$
	ΔD	—	0.00	0.00	0.01	0.01	Gy
CTV2	$D_{99\%}$	> 90	-0.33	-0.32	-0.58	-0.86	$\%D_{pres}$
	$D_{95\%}$	> 95	-0.22	-0.07	-0.60	-0.41	$\%D_{pres}$
	$D_{5\%}$	ALARP	-6.10	-5.95	-5.98	-5.75	$\%D_{pres}$
	$D_{2\%}$	ALARP	-6.10	-5.96	-5.85	-5.74	$\%D_{pres}$
Brainstem	D_{1cc}	< 54					
	$D_{0.1cc\%}$	< 55					
0.00	0.00	0.00	0.00	Gy			
-0.20	-0.20	-0.23	-0.24	Gy			
Spinal cord	D_{1cc}	< 48	0.00	0.00	0.00	0.00	Gy
	$D_{0.1cc\%}$	< 46	-0.38	-0.38	-0.36	-0.37	Gy
Parotid Ip	D_{mean}	ALARP	0.00	0.00	-0.01	-0.01	Gy
Parotid Con	D_{mean}	< 20	0.00	0.00	0.00	0.00	Gy
SMG Ip	D_{mean}	ALARP	0.00	0.01	0.00	0.00	Gy
SMG Con	D_{mean}	< 20	**0.54**	**0.54**	**-0.02**	**-0.03**	Gy

Table S13: Percentage pass rates of voxels within each ROI from the oropharyngeal O1 under gamma analysis comparing dose distributions from single and dual arc PAT under step-and-shoot delivery to the corresponding plan under continuous delivery with and without applying the random angular and positional shifts from a Gaussian probability distribution with $\sigma = 0.33$ mm/° to each spot. Abbreviations: DD = dose-difference, DTA = distance-to-agreement, CTV = clinical target volume, SMG = sub-mandibular gland, Ip = ipsilateral, Con = contralateral.

ROI	DD/DTA Criteria	Single arc PAT		Dual arc PAT	
		Original	Shifted	Original	Shifted
CTV1	1%/1mm	99.6	98.5	92.4	100.0
	3%/3mm	100.0	100.0	100.0	100.0
CTV2	1%/1mm	98.8	98.0	81.8	100.0
	3%/3mm	100.0	100.0	100.0	99.9
Brainstem	1%/1mm	97.1	95.5	81.4	100.0
	3%/3mm	100.0	100.0	100.0	100.0
Spinal cord	1%/1mm	100.0	99.5	81.3	100.0
	3%/3mm	100.0	100.0	100.0	100.0
Parotid Ip	1%/1mm	100.0	100.0	99.3	100.0
	3%/3mm	100.0	100.0	100.0	100.0
Parotid Con	1%/1mm	98.8	97.5	99.3	100.0
	3%/3mm	100.0	100.0	100.0	100.0
SMG Ip	1%/1mm	100.0	100.0	96.6	100.0
	3%/3mm	100.0	100.0	100.0	100.0
SMG Con	1%/1mm	96.1	96.2	69.0	100.0
	3%/3mm	100.0	100.0	100.0	100.0

4.3.6 Oropharyngeal O2

Table S14: Comparison of the dose metric change in the nominal scenario during each emulated delivery technique relative to the original step-and-shoot PAT plan for the oropharyngeal O2 dataset. Bold text indicates a failure of clinical tolerance. Abbreviations: CTV = clinical target volume, SMG = sub-mandibular gland, Ip = ipsilateral, Con = contralateral, ALARP = as low as reasonably possible.

ROI	Metric	Clinical goal	Single arc PAT		Dual arc PAT		Units
			Original	Shifted	Original	Shifted	
CTV1	$D_{99\%}$	> 90	-0.48	-0.19	-1.81	-1.62	$\%D_{pres}$
	$D_{95\%}$	> 95	-0.53	-0.39	-1.38	-1.30	$\%D_{pres}$
	$D_{5\%}$	≤ 105	0.29	0.32	1.87	2.11	$\%D_{pres}$
	$D_{2\%}$	≤ 107	0.45	0.34	2.64	2.77	$\%D_{pres}$
	ΔD	—	0.02	0.02	0.07	0.07	Gy
CTV2	$D_{99\%}$	> 90	**-5.71**	**-5.87**	-3.25	-3.28	$\%D_{pres}$
	$D_{95\%}$	> 95	-0.51	-0.51	-2.34	-2.36	$\%D_{pres}$
	$D_{5\%}$	ALARP	-6.14	-6.19	-6.00	-6.02	$\%D_{pres}$
	$D_{2\%}$	ALARP	-6.33	-6.28	-5.97	-5.87	$\%D_{pres}$
Brainstem	D_{1cc}	< 54	-0.04	-0.04	0.08	0.08	Gy
	$D_{0.1cc\%}$	< 55	-0.58	-0.58	-0.16	-0.16	Gy
Spinal cord	D_{1cc}	< 48	0.00	0.00	0.02	0.02	Gy
	$D_{0.1cc\%}$	< 46	-0.39	-0.38	-0.31	-0.31	Gy
Parotid Ip	D_{mean}	ALARP	0.02	-0.03	0.02	0.02	Gy
Parotid Con	D_{mean}	< 20	-0.03	-0.03	0.02	0.02	Gy
SMG Ip	D_{mean}	ALARP	0.00	0.00	0.03	0.03	Gy
SMG Con	D_{mean}	< 20	**-0.08**	**-0.08**	**0.15**	**0.15**	Gy

Table S15: Percentage pass rates of voxels within each ROI from the oropharyngeal O2 case under gamma analysis comparing dose distributions from single and dual arc PAT under step-and-shoot delivery to the corresponding plan under continuous delivery with and without applying the random angular and positional shifts from a Gaussian probability distribution with $\sigma = 0.33$ mm/° to each spot. Abbreviations: DD = dose-difference, DTA = distance-to-agreement, CTV = clinical target volume, SMG = sub-mandibular gland, Ip = ipsilateral, Con = contralateral.

ROI	DD/DTA Criteria	Single arc PAT		Dual arc PAT	
		Original	Shifted	Original	Shifted
CTV1	1%/1mm	68.7	68.1	48.8	47.4
	3%/3mm	99.8	99.8	97.4	97.2
CTV2	1%/1mm	60.4	60.6	45.1	45.8
	3%/3mm	99.4	99.4	93.4	93.6
Brainstem	1%/1mm	56.4	56.7	36.9	36.1
	3%/3mm	96.7	97.9	93.2	93.2
Spinal cord	1%/1mm	64.5	64.9	41.7	41.5
	3%/3mm	100.0	100.0	97.7	98.0
Parotid Ip	1%/1mm	57.4	55.6	35.7	33.7
	3%/3mm	100.0	100.0	99.2	99.0
Parotid Con	1%/1mm	53.6	53.1	39.2	39.3
	3%/3mm	100.0	99.9	99.7	99.7
SMG Ip	1%/1mm	83.4	81.6	44.8	45.8
	3%/3mm	100.0	100.0	98.6	74.0
SMG Con	1%/1mm	1.7	2.3	1.1	1.0
	3%/3mm	100.0	100.0	73.4	74.0

References

[1] N. G. Burnet, R. I. Mackay, E. Smith, A. L. Chadwick, G. A. Whitfield, D. J. Thomson, M. Lowe, N. F. Kirkby, A. M. Crellin, and K. J. Kirkby, "Proton beam therapy: perspectives on the national health service england clinical service and research programme," *The British Journal of Radiology*, vol. 93, no. 1107, p. 20190873, 2020.

[2] A. H. Aitkenhead, P. Sitch, J. C. Richardson, C. Winterhalter, I. Patel, and R. I. Mackay, "Automated Monte-Carlo re-calculation of proton therapy plans using GEANT4/GATE: implementation and comparison to plan-specific quality assurance measurements," *The British Journal of Radiology*, vol. 93, no. 1114, p. 20200228, 2020.

[3] L. Grevillot, D. Boersma, H. Fuchs, A. Aitkenhead, A. Elia, M. Bolsa, C. Winterhalter, M. Vidal, S. Jan, U. Pietrzyk, *et al.*, "GATE-RTion: a GATE/GEANT4 release for clinical applications in scanned ion beam therapy," *Medical Physics*, vol. 47, no. 8, pp. 3675–3681, 2020.
